# Effect of Morning and Evening Exercise on Energy Balance: A Pilot Study

**DOI:** 10.3390/nu14040816

**Published:** 2022-02-15

**Authors:** Seth A. Creasy, Liza Wayland, Shelby L. Panter, Sarah A. Purcell, Rebecca Rosenberg, Erik A. Willis, Bethelhem Shiferaw, Laura Grau, Matthew J. Breit, Daniel H. Bessesen, Edward L. Melanson, Victoria A. Catenacci

**Affiliations:** 1 Division of Endocrinology, Metabolism, and Diabetes, University of Colorado Anschutz Medical Campus, Aurora, CO 80045, USA; shelby.panter@cuanschutz.edu (S.L.P.); sarah.purcell@cuanschutz.edu (S.A.P.); rebecca.rosenberg@cuanschutz.edu (R.R.); matthew.breit@cuanschutz.edu (M.J.B.); daniel.bessesen@cuanschutz.edu (D.H.B.); ed.melanson@cuanschutz.edu (E.L.M.); vicki.catenacci@cuanschutz.edu (V.A.C.); 2 Anschutz Health and Wellness Center, University of Colorado Anschutz Medical Campus, Aurora, CO 80045, USA; liza.wayland@cuanschutz.edu; 3 Center for Health Promotion Disease Prevention, University of North Carolina-Chapel Hill, Chapel Hill, NC 27599, USA; erik.willis@unc.edu; 4 Department of Nutrition, Gillings School of Global Public Health, University of North Carolina-Chapel Hill, Chapel Hill, NC 27599, USA; 5 Department of Biostatistics and Informatics, Colorado School of Public Health, University of Colorado Anschutz Medical Campus, Aurora, CO 80045, USA; bethelhem.shiferaw@cuanschutz.edu (B.S.); laura.grau@cuanschutz.edu (L.G.); 6 Division of Endocrinology, Denver Health Medical Center, Denver, CO 80204, USA; 7 Eastern Colorado VA Geriatric Research, Education, and Clinical Center, Aurora, CO 80045, USA; 8 Division of Geriatrics, University of Colorado Anschutz Medical Campus, Aurora, CO 80045, USA

**Keywords:** obesity, weight loss, exercise timing, diurnal

## Abstract

The purpose of this study was to evaluate the feasibility and acceptability of randomizing adults with overweight and obesity (BMI 25–40 kg/m^2^) to morning (06:00–10:00) or evening (15:00–19:00) aerobic exercise. Participants completed four exercise sessions per week in the morning (AM, *n* = 18) or evening (PM, *n* = 15). The exercise program was 15 weeks and progressed from 70 to 80% heart rate maximum and 750–2000 kcal/week. Bodyweight, body composition, total daily energy expenditure (TDEE), energy intake (EI), sleep, sedentary behavior (SB), non-exercise physical activity (NEPA), and maximal aerobic capacity were assessed at baseline and week 15. Study retention was 94% and adherence to the supervised exercise program was ≥90% in both groups. Weight change was −0.9 ± 2.8 kg and −1.4 ± 2.3 kg in AM and PM, respectively. AM and PM increased TDEE (AM: 222 ± 399 kcal/day, PM: 90 ± 150 kcal/day). EI increased in AM (99 ± 198 kcal/day) and decreased in PM (−21 ± 156 kcal/day) across the intervention. It is feasible to randomize adults with overweight and obesity to morning or evening aerobic exercise with high levels of adherence. Future trials are needed to understand how the timing of exercise affects energy balance and body weight regulation.

## 1. Introduction

Well-designed studies have demonstrated that mean weight loss from exercise interventions is less than predicted based on the energy expenditure (EE) of exercise sessions [1,2,3,4,5]. In addition, there is substantial interindividual variability in the weight loss response to exercise interventions. Both attenuated weight loss and weight loss variability have been attributed to compensatory mechanisms such as reductions in other components of EE and/or increases in energy intake (EI) [1,6,7,8,9,10,11,12]. The timing (i.e., time of day) of exercise may affect these compensatory responses and the weight loss response [13,14,15,16,17,18,19,20,21,22,23]. In addition, exercise timing may influence factors related to overall adherence and engagement in exercise, thereby influencing body weight [24]. However, there are limited studies examining the effect of exercise at different times of the day on components of EE, EI, and weight loss.

Evidence suggests that the time of day in which exercise is performed may impact weight loss [13,14,16,17]. A secondary analysis by Willis et al. found that young adults with overweight and obesity who completed high amounts of supervised aerobic exercise (2000–3000 kcal/week) in the morning lost significantly more weight compared to those who completed the same amount of exercise in the evening [13]. Alizadeh et al. also found a beneficial effect of morning aerobic exercise on body weight as compared to evening exercise across a 6-week exercise intervention [14]. In contrast, others have found that evening exercise results in greater decreases in fat mass compared to morning exercise [16,17]. A recent randomized trial found no differences in weight or body composition change following 12 weeks of morning versus evening combined aerobic and resistance exercise [23]. All the above studies have been limited by post hoc analyses, small sample sizes, or exercise interventions that were not specifically designed for weight loss. In addition, these studies lacked objective measures of energy balance. Thus, a prospective, randomized trial is needed to understand the effects of morning versus evening exercise on EE, EI, and weight loss in individuals with overweight and obesity [18]. Prior to a large-scale study, data on the feasibility and acceptability of randomizing adults to morning or evening aerobic exercise are needed.

The primary purpose of this study was to evaluate the feasibility and acceptability of randomizing individuals with overweight and obesity to complete 2000 kcal/week of aerobic exercise in the morning (06:00–10:00) versus evening (15:00–19:00). In addition, measurements of change in body composition, energy balance (i.e., EE and EI), and other compensatory responses were assessed over the 15-week intervention.

## 2. Materials and Methods

### 2.1. Participants

Volunteers were recruited to participate in this study by using email announcements and community flyers from February 2020 to October 2020. All study procedures were approved by the Colorado Multiple Institutional Review Board. This study was registered at clinicaltrials.gov (accessed on 12 October 2021) (NCT04262115). Interested individuals first contacted study staff to express interest in the study, then completed an online questionnaire to determine preliminary eligibility. All participants provided written informed consent prior to participation in any study procedures. After determining initial eligibility, participants underwent a physical examination with a licensed medical provider and completed a health history form and a blood draw to determine if they met all eligibility criteria. Primary inclusion criteria were age 18–56 years old; body mass index (BMI) of 25.0–40.0 kg/m^2^; physically inactive (defined as <150 min/week of moderate intensity physical activity over the past 3 months); owning a cell phone to complete text messaging surveys; and possessing a willingness to accept their randomized group assignment. Individuals were excluded if they had any physical or medical conditions that would preclude safely engaging in the exercise intervention, including significant cardiovascular disease, certain types of cancer, metabolic diseases, musculoskeletal, neurological, and psychiatric disorders. Individuals were also excluded for nicotine use; taking medications known to affect appetite, metabolism, or body weight; prior weight loss surgery; irregular sleep/wake patterns that would hinder adherence to the exercise intervention; current dieting or planning to diet during the study; eating disorders; and weight change >5% in the past 3 months. Females who were currently pregnant, lactating, or pregnant within the past 6 months were also excluded.

### 2.2. Supervised Exercise Interventions

Participants were stratified by sex and baseline BMI and randomized within strata in a 1:1 ratio to complete 15 weeks of supervised aerobic exercise in the morning (AM; 06:00–10:00) or the evening (PM; 15:00–19:00). Participants had to complete 3 sessions per week “in-person” under direct supervision of study staff and 1 session per week “on-own.” In-person sessions were to be completed Monday to Friday during the defined time windows. On-own sessions were to take place during the defined time window but could take place any day of the week and at any location. Supervised in-person exercise sessions were completed in the Anschutz Health and Wellness Center (AHWC) where participants had access to treadmills, stationary upright cycles, recumbent cycles, ellipticals, stair stepping machines, and rowing machines. This study occurred during the SARS-CoV-2 pandemic; thus, all participants were required to wear a mask or face covering during exercise sessions at the AHWC as per local public health guidelines. Exercise progressed from moderate (70% heart rate maximum) to vigorous (80% heart rate maximum) intensity and from 187.5 kcal/session (750 kcal/week) to 500 kcal/session (2000 kcal/week) by week 11 (Table 1). Since weight change was one of the primary outcomes of this study, we elected to control exercise EE rather than prescribing exercise based on minutes per day, which could result in different energy deficits between participants. Each participant was provided an individualized time target to expend the prescribed amount of kilocalories per session. These prescriptions were based on measured exercise EE at 70%, 75%, and 80% heart rate maximum during the baseline maximal aerobic capacity test. For example, if a participant expended 9.3 kcal/min at 80% of their heart rate maximum, they would need to exercise for 54 min per session (500 kcal ÷ 9.3 kcal/min = 54 min). Participants wore Polar H10 heart rate monitors for in-person and on-own sessions and utilized the Polar Beat smartphone application to record exercise session data. Study staff verified that each exercise session was completed at the prescribed heart rate and duration. Participant attendance and adherence to the exercise prescription were tracked throughout the study, and participants received an email every two weeks with their attendance and adherence to the exercise prescription. Attendance and adherence were calculated weekly and averaged across the exercise intervention. Attendance (%) was calculated as the total number of exercise sessions completed divided by total number of exercise sessions prescribed (60 sessions). Adherence (%) was calculated as the total number of minutes in the target heart rate zone each week divided by the total number of minutes prescribed each week. Participants were instructed to eat ad libitum and not initiate major dietary changes (e.g., intentional caloric restriction, intermittent fasting, vegetarian diet, vegan diet, etc.) throughout the course of the study. At week 15, participants completed a multicomponent survey on several aspects of the exercise intervention.

Participants were provided with one-on-one behavioral support during weeks 2, 6, 10, and 14 with the overall goal of promoting adherence to the exercise prescription. These sessions were grounded in the Social Cognitive Theory [25] and included topics such as goals and planning, time management, feedback and monitoring, and motivation. Sessions were provided via Zoom (Zoom Video Communications Inc., San Jose, CA, USA), led by trained study staff, and lasted approximately 30 min in duration.

### 2.3. Body Weight and Composition

Body weight was measured at baseline and week 15 using a calibrated digital scale. Participants wore a gown during weight measurements. Fat mass (FM) and fat-free mass (FFM, lean tissue + bone) were also measured at baseline and week 15 using dual-energy X-ray absorptiometry (DXA, Hologic Discovery W, Bedford, MA, USA). DXA scans were performed and analyzed by trained members of the Colorado Clinical and Translational Research Center.

### 2.4. Sleep, Sedentary Behavior, and Physical Activity

All participants wore the activPAL^TM^ v4 (AP; PAL Technologies, Glasgow, UK) and Actiwatch 2 (AW2; Philips Respironics, Bend, OR, USA) for 14 consecutive days at baseline and during the final two weeks of the exercise intervention (weeks 14–15). The AP is a thigh-worn accelerometer that can be used to detect posture (lying, sitting, and standing), sedentary behavior (SB; <1.5 metabolic equivalents), and physical activity (≥1.5 metabolic equivalents). Sleep duration, bedtime, and waketime were determined using the AW2 and a standardized approach utilizing a combination of event markers, the sleep/wake log, activity levels, and light sensor data [26] (Actiware version 6.0.9, Philips Respironics, Bend, OR, USA). For a day to be considered valid, participants must have worn the devices for 24 h. Only days with valid data from both devices were analyzed. Only participants with at least 7 valid days (≥2 weekend days, ≥5 weekdays) were included in the analysis. Time spent asleep (as detected by the AW2) and time spent exercising (using the exercise intervention logs) were manually removed so that SB and non-exercise physical activity (NEPA) could be calculated.

### 2.5. Components of Energy Balance

Resting energy expenditure (REE) and total daily energy expenditure (TDEE) were measured in a subset of 23 participants (*n* = 12 for AM, *n* = 11 for PM) at baseline and week 15. REE was measured by using indirect calorimetry (Truemax 2400, Parvomedics, Salt Lake City, UT, USA) and the ventilated hood technique. Prior to each REE measurement, the metabolic cart’s gas analyzers and flow meter were calibrated according to the manufacturer’s recommendations. REE was measured in the morning after a ≥12 h fast. Participants rested supine for 30 min prior to data collection. Data were collected for 15 min while the participant remained in a supine position in a dark room under thermoneutral conditions. The last 10 min of data were averaged after inspection and exclusion of data that did not meet quality control measures (minute-by-minute coefficient of variation < 10% for VO_2_, VCO_2_, respiratory quotient, and REE). TDEE was measured using the doubly labeled water (DLW) technique. Participants were dosed with ~1.1 g/kg of body weight of 10% atom percent enriched (APE) ^18^O-labeled water and ~0.08 g/kg of body weight of 99.8% APE ^2^H-labeled water. Urine samples were collected prior to dosing and 4 h, 5 h, 7 days, and 14 days after dosing. An amount of 10 mL of each sample was immediately pipetted into internally threaded cryovials and stored at −80 °C until analysis. Samples were analyzed in the University of Colorado Doubly Labeled Water Isotope Core using off-axis integrated cavity output spectroscopy (ABB Inc., San Jose, CA, USA) [27]. VCO_2_ was calculated using the intercept method and the equation of Speakman et al. [28]. TDEE was calculated using the Weir equation assuming a respiratory quotient (RQ) of 0.86 and averaged over the observation period [29]. For follow-up, the DLW assessment was conducted during weeks 14–15 (i.e., the last two weeks of exercise). Non-exercise energy expenditure (NEEE) was calculated as TDEE- REE- 286 kcal (exercise energy expenditure; 2000/7 = 286 kcal/day). Mean EI over the course of the of the exercise intervention was calculated using the intake-balance method in this same subset of participants [30]. The intake-balance method utilizes change in body stores (∆FM = 9.3 kcal/g, ∆FFM = 1.1 kcal/g) measured via DXA and TDEE measured using DLW to calculate EI. TDEE can be considered as an adequate estimate for EI when participants are weight stable; thus, baseline TDEE was considered to be equal to EI. For mean EI across the intervention, we used change in body stores from DXA and an assumed a TDEE of (½ Baseline TDEE + ½ 15-week TDEE). This TDEE equation was selected to account for the exercise ramp up for weeks 1–10.

### 2.6. Cardiorespiratory Fitness

Cardiorespiratory fitness was assessed at baseline and week 15 using a maximal effort aerobic capacity test with a modified Balke treadmill protocol. Expired gases were collected and analyzed at 20-s intervals using standard indirect calorimetry (Truemax 2400, Parvomedics, Salt Lake City, UT, USA). Participants were asked to continue exercising until volitional exhaustion.

### 2.7. Statistical Analyses

Statistical analyses were performed using SAS 9.4 (SAS Institute Inc., Cary, NC, USA). Baseline characteristics are presented as mean ± standard deviation (SD) and *n* (%) by group. Changes in weight, body composition, EE, EI, sleep duration, SB, NEPA, and blood markers are presented as mean ± SD. Descriptive statistics were used to summarize enrollment, retention, adherence, and acceptability data. Since this was a pilot study, no formal between-group comparisons were made, as is recommended for pilot studies [31,32,33]. This study was not designed or powered to detect statistically significant differences between groups; thus, all findings are considered preliminary.

## 3. Results

### 3.1. Enrollment and Retention

**Figure 1:**
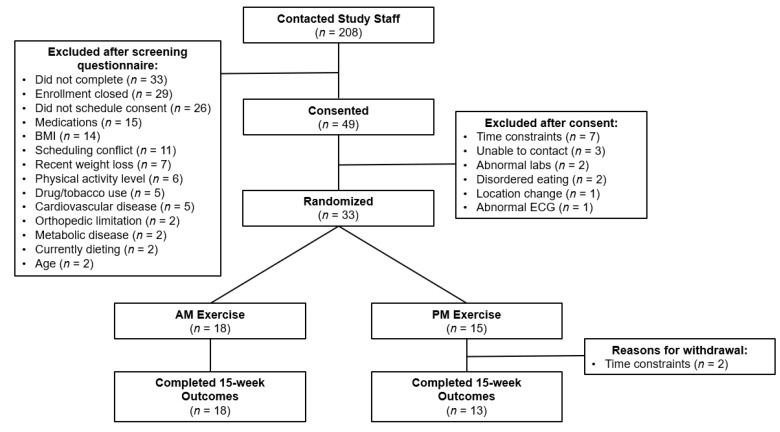
Study enrollment.

A total of 208 people contacted study staff with interest in the study (Figure 1). Of those, 146 completed the online eligibility questionnaire, and 75 met the preliminary eligibility criteria. A total of 36 people consented and completed in-person screening measures. Three people (8%) were determined to be ineligible based on the screening visit. Baseline characteristics of randomized participants are shown in Table 2. Age, BMI (kg/m^2^), and weight were similar at baseline. Overall retention at week 15 was 94% (31/33). Two participants (13%) in the PM group withdrew from the study due to time constraints and an inability to continue participating in supervised exercise sessions; no participants withdrew from the AM group. Of the participants that completed the study, the primary motivations for enrolling included weight loss (42%), general health benefits (42%), complimentary gym membership (6%), health assessment information (6%), and an overall interest in contributing to research (3%).

### 3.2. Exercise Intervention Adherence

**Figure 2:**
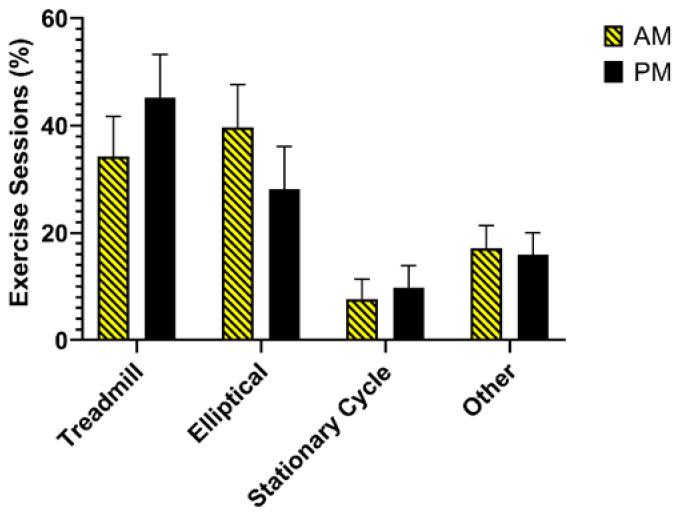
Exercise equipment use.

Exercise intervention adherence data are presented in Table 3. AM and PM had similar levels of attendance and adherence across the intervention. Overall attendance of the prescribed exercise sessions was 90 ± 8% and 91 ± 10% in AM and PM, respectively, and overall adherence to the prescribed exercise program was 90 ± 9% and 91 ± 10% in AM and PM, respectively. Overall adherence ranged from 70 to 100% in AM and 71–100% in PM. AM and PM had high levels of attendance and adherence across the exercise ramp-up (weeks 1–10). There were slight decreases in both attendance and adherence at the full prescription (2000 kcal/week), but both remained >80%. Compliance to the prescribed exercise duration and intensity was nearly identical to prescriptions and was similar between groups. Over the course of the intervention, 31% of AM started exercise sessions between 06:00 and 06:59, 23% started between 07:00 and 07:59, 24% started between 08:00 and 8:59, 18% started between 09:00 and 9:59, and 4% started outside of their prescribed time. Twenty-five percent of PM started their exercise sessions between 15:00 and 15:59, followed by 23% who started between 16:00 and 16:59, 30% started between 17:00 and 17:59, 13% started between 18:00 and 18:59, and 9% started outside of their prescribed time. Equipment use for exercise sessions is shown in Figure 2. For PM, the treadmill was used most frequently, followed by the elliptical, other, and stationary cycle. For AM, the elliptical was used most frequently followed by the treadmill, other, and stationary cycle. AM and PM increased both relative aerobic capacity (AM: 2.6 ± 1.7 mL/kg/min, PM: 3.6 ± 3.0 mL/kg/min) and absolute aerobic capacity (AM: 0.20 ± 0.12 L/min, PM: 0.26 ± 0.40 L/min) at week 15.

### 3.3. Exercise Intervention Acceptability

Ninety-four percent of participants reported that they enjoyed the supervised exercise program. Eighty-one percent of participants reported that the number of exercise sessions per week (4) and the number of supervised sessions per week (3) were the right amount while the other 19% of participants thought it was too much. Fifty-five percent of participants reported that the duration of each exercise session was the right amount while the other 45% reported that the sessions were too long. Fifty-two percent of participants reported that the intensity of the exercise at the beginning of the study was too easy, 45% reported that it was appropriate, and 3% found it too difficult. At the end of the study, 91% reported that the intensity was appropriate, 6% reported it was too easy, and 3% reported it was too difficult. All participants (100%) liked the ability to complete on-own sessions each week and the primary locations for those on-own sessions include at the AHWC (45%), at home (29%), outdoors (16%), and at another exercise facility (10%). Twenty-nine percent of participants reported being intimidated when starting exercise for the first time. Eighty percent of participants reported being comfortable with exercise after 2 weeks, 6% reported being comfortable after 4 weeks, 10% after 8 weeks, and 3% were never comfortable. Seventy-seven percent of participants reported that the one-on-one behavioral support sessions were helpful. During the 15-week intervention, 15 adverse events were reported. Of those, four were classified as “definitely related” or “probably related” to the intervention.

### 3.4. Change in Weight and Body Composition

**Figure 3:**
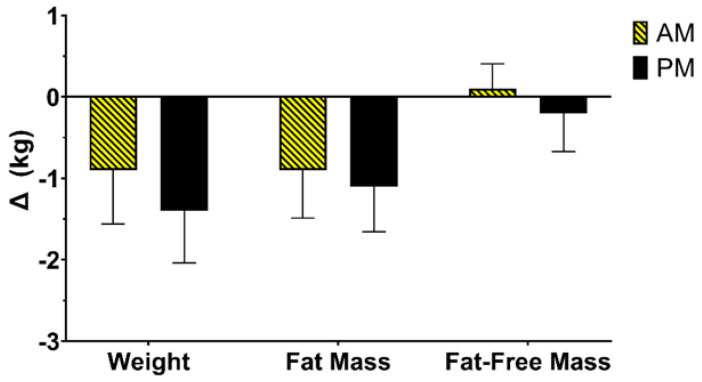
Change in weight and body composition.

AM (−0.9 ± 2.8 kg) and PM (−1.4 ± 2.3 kg) both lost a modest amount of weight during the intervention (Figure 3). Changes in weight were primarily driven by FM loss for both AM (−0.9 ± 2.5 kg) and PM (−1.4 ± 2.3 kg); however, PM showed a slight decrease in FFM.

### 3.5. Change in Energy Expenditure and Energy Intake

Twenty-three participants (AM: *n* = 12, PM: *n* = 11) completed the DLW protocol at baseline and during weeks 14–15 of the exercise intervention. AM and PM both increased TDEE across the intervention (Table 4). Resting EE remained similar in both groups from baseline to week 15, and NEEE decreased in both AM and PM across the intervention. Baseline EI was assumed to be equivalent to baseline TDEE as participants were weight stable across the 14 day DLW assessment period (0.24 ± 0.71 kg). EI across the intervention increased in AM but decreased in PM.

### 3.6. Change in Sleep, Sedentary Behavior, and Physical Activity

Both groups had minimal changes in NEPA at week 15 (AM: −6.8 ± 28.6 min/day, PM: 7.4 ± 46.2 min/day). Both groups decreased SB at week 15 (AM: −36.3 ± 75.6 min/day, PM: −88.5 ± 137.2 min/day). AM exhibited a decrease in sleep duration (−10.2 ± 42.6 min/day), while PM increased sleep duration (26.6 ± 73.0 min/day) at week 15.

## 4. Discussion

Mean weight loss from exercise alone (i.e., without a dietary intervention) is often less than expected based on the EE of the exercise due to compensatory mechanisms [1,2,3,4,5]. Until recently, the time-of-day exercise is performed and its potential impact on weight loss and energetic compensation has been underappreciated. Accumulating evidence suggests that the timing of exercise may affect changes in weight, body composition, EI, and EE [18]. However, studies to date have significant limitations, including secondary analyses, small sample sizes, limited populations, various exercise stimuli, and limited objective outcome measures [13,14,15,16,17,22,23,34]. These limitations have made it difficult to make comparisons across studies and draw conclusions about the best time of time of day to exercise for weight loss. The current study begins to address these gaps by including a randomized design, an aerobic exercise intervention based on exercise EE, and objective measurements of EE, EI, and non-exercise behaviors. This study also provides recruitment feasibility data and information on intervention adherence and acceptability that may inform future studies. Our primary findings include the following: (1) adults with overweight and obesity can adhere to high amounts of prescribed aerobic exercise in the morning and evening, (2) AM and PM lost similar amounts of weight and fat mass, and (3) the timing of exercise may differentially affect components of energy balance. Adequately powered studies are needed to confirm these preliminary findings.

This study had similar study interest rates compared to other pilot studies utilizing supervised aerobic exercise [35,36,37,38], demonstrating that the timing of exercise does not appear to significantly hinder interest. We found that, of the individuals who filled out the screening questionnaire, ~40% were eligible. The consent rate (i.e., number of people who consented ÷ number of people eligible based on initial screen) was 63%, which was similar to another pilot study of AM vs. PM exercise (59%) [15]. There were a significant number of subjects who consented to participate in this study but did not attend the in-person screening visit (*n* = 13). This may have been due to delays between the consent date and the in-person screening visit that were due to a campus-wide research shutdown during the Sars-CoV-2 pandemic. We found that a high number of participants who were eligible based on the screening questionnaire remained eligible after the in-person screening visit (92%), suggesting that the online screening tool used in this study was highly effective at identifying ineligible participants. This study had a high retention rate (94%), which is similar to other randomized, supervised exercise studies using the morning and evening exercise paradigm [15,22].

Both AM and PM exhibited high levels of adherence across the 15-week exercise intervention. Adherence to the number of completed sessions per week, exercise EE (kcal/week), and exercise intensity were the highest during weeks 1–10. During weeks 11–15, when the prescription reached 2000 kcal/week, both AM and PM demonstrated decreases in adherence; however, overall adherence remained high (>80%). These levels of exercise adherence are similar to or greater than other supervised exercise interventions utilizing morning and evening exercise [15,16,17,22]. In the one other randomized study utilizing aerobic exercise only, adherence to supervised exercise sessions was 94% in the morning exercise group and 87% in the evening exercise group over a 12-week intervention [15]. Most participants reported that the number of total and supervised sessions per week was the right amount, which may have helped to increase adherence. Nearly half of participants reported that exercise durations were too long, which may have been the reason that adherence declined during weeks 11–15, when prescriptions were the longest. Participants did report that exercise intensity was the right amount during weeks 8–15. This is important, as this study utilized an aerobic exercise prescription that progressed to vigorous intensity. There were four adverse events that were definitely or probably related to the exercise intervention. In all cases, participants were able to continue exercising with modifications (e.g., rest, ice, walking instead of running, switching to elliptical or stationary cycle). Our data suggest that participants with overweight or obesity can safely engage in high amounts of vigorous exercise (80% heart rate maximum) when the exercise ramp-up period is appropriate.

Both AM and PM lost modest amounts of weight across the 15-week intervention. Prior short-term studies confirm that weight loss from morning and evening exercise, without dietary modification, results in modest mean weight loss of 1–2 kg [14,15,16,17,23]. Alizadeh et al. found that a 6-week aerobic exercise program completed from 08:00 to 10:00 resulted in greater weight loss than if it was competed between 14:00 and 16:00 [14]. However, other short-term studies have failed to observe significant differences in change in body weight in response to timed exercise [16,17,23]. In a longer-term study, Willis et al. found that 10 months of a high volume of aerobic exercise completed in the morning resulted in clinically significant weight loss [13]. In that study, morning exercisers lost significantly more weight (−7.2 ± 1.2%) compared to evening exercisers (−2.1 ± 1.0%). There are several possible explanations for these discordant findings. First, morning and evening exercise have been defined differently by each study. Furthermore, the timing of exercise has been based on clock time rather than based on an individual’s biological clock (e.g., dim-light melatonin onset or offset) or chronotype. It is possible that individual circadian timing or individual chronotype may modify the relationship between exercise timing and weight loss. In addition, prescribing exercise based on a biological indicator of time may produce a more homogenous metabolic response. Complicating things further, each of these studies have enrolled different populations. Individual-level factors such as sex, race/ethnicity, age, BMI, and disease status may influence body weight response. Finally, in each of these studies, the exercise stimulus and duration have varied. Future studies enrolling similar populations and utilizing similar exercise protocols are needed to understand if there is an optimal time of day to exercise for weight loss. In addition, an adequately powered, randomized trial with a longer exercise intervention (≥ 6 months) is warranted.

This study found that the morning and evening exercise may differentially affect sleep duration and sleep timing. PM increased sleep duration (~30 min/day) and AM decreased sleep duration (~10 min/day). Individuals performing AM exercise appeared to sleep earlier at night, but also woke up earlier to engage in exercise. Individuals performing PM exercise appeared to sleep earlier at night and wake up later during the exercise intervention. Thomas et al. found that individuals engaging in evening exercise went to bed later and woke up later than individuals engaging in morning exercise [39]. In that study, evening exercisers also slept for ~30 min more than morning exercisers, although this was not statistically significant. In contrast to the changes in sleep, we found that both AM and PM exhibited minimal changes in SB and NEPA during the exercise intervention. This lack of behavioral compensation contrasts the findings of Willis et al., where morning exercisers maintained NEPA and decreased SB, but evening exercises had slight decreases in NEPA and increases in SB [13]. Typically, following the initiation of an exercise routine, there is some level of behavioral compensation outside of the exercise sessions [6]. The time of day of exercise may affect this compensation. For example, exercise training in the morning may result in fatigue and, thus, decreased physical activity the remainder of the day. However, these pilot data do not support this hypothesis.

In our subgroup of participant with DLW measurements, we found that AM and PM had similar overall changes in energy balance but the effects on TDEE and EI differed. AM exhibited increases in TDEE that were consistent with the prescribed exercise, while PM had an attenuated increase in TDEE. Furthermore, AM increased EI by ~100 kcal/day, but PM had a slight decrease in EI (20 kcal/day). Willis et al. also found that morning exercise resulted in an increase in TDEE similar to what was expected based on exercise EE and evening exercise resulted in a blunted EE response [13]. It is possible that morning exercise results in a phase advance thereby promoting circadian alignment with the light–dark cycle [39]. It is unclear whether circadian alignment promotes a more robust metabolic response to exercise; however, this is certainly possible. Circadian misalignment has been shown to result in a decrease in total EE under controlled laboratory conditions [40]. Willis et al. found that morning exercise resulted in a nonsignificant decrease in EI, whereas, evening exercise resulted in a nonsignificant increase in EI. In a recent randomized pilot study, morning exercise and evening exercise resulted in similar decreases in self-reported EI over 12 weeks [23]. In that study, morning exercise also resulted in greater increases in perceived fullness, while evening exercise resulted in a greater decrease in disinhibition. These inconsistent findings require further investigation. It is also possible that exercise at different times of the day may have differential effects on appetite-related hormones and their patterns across the day. While the effects are not consistent, exercise has been shown to alter appetite-related hormones and subsequent energy intake [41,42]. Controlled, laboratory studies are needed to understand how the timing of exercise may modify the relationship between exercise and appetite regulation. In addition, future studies should evaluate the potential importance of chronotype, circadian alignment, sex, and other individual factors that could influence the interaction between exercise timing and energy balance.

There are several limitations to this pilot study. Our study was not powered nor designed to detect meaningful differences between AM and PM; thus, results should be interpreted with caution. The duration of the exercise intervention was 15 weeks, with the target prescription (2000 kcal/week) only lasting 5 weeks. While this prescription is sufficient to produce weight loss, the duration of the prescription may not have been long enough to result in meaningful changes in body weight or compensatory responses. Furthermore, exercise EE was only measured at baseline in this study. It was not feasible to test and adjust exercise EE during the study to account for changes in fitness and body composition; thus, it is possible that the exercise prescriptions were increasingly inaccurate as the study progressed. Only a subset of participants completed the DLW assessment of TDEE and EI. It is possible that the results of this subset (*n* = 23) are not representative of the entire cohort (*n* = 33). Furthermore, assessments of TDEE, SB, NEPA, and sleep were conducted over a two-week period at baseline and during weeks 14–15 of the exercise intervention. These assessment periods may not be reflective of behavior and energy balance over the course of the exercise intervention. Finally, this study was conducted during the SARS-CoV-2 pandemic, and it is possible that adherence to the exercise intervention is not reflective of typical exercise adherence.

Adults with overweight and obesity were able to complete aerobic exercise progressing to vigorous intensity and 2000 kcal/week prescribed in the morning and the evening with high levels of adherence. Reductions in weight and fat mass were modest and similar between the morning and evening exercise groups. The timing of exercise may affect energy balance regulation and non-exercise behaviors such as sleep. A large-scale randomized trial is needed to examine the effect of morning versus evening aerobic exercise on weight loss. In addition, controlled studies of morning and evening exercise are needed to understand mechanisms through which exercising timing results in differential energetic perturbations.

## Figures and Tables

**Table 1:** Exercise intervention progression.

Week	Sessions/Week	Kcal/Session	Intensity	Kcal/Week
1–2	3 supervised, 1 on-own	187.5	70% HRmax	750
3–4	3 supervised, 1 on-own	250	70% HRmax	1000
5–6	3 supervised, 1 on-own	312.5	75% HRmax	1250
7–8	3 supervised, 1 on-own	375	75% HRmax	1500
9–10	3 supervised, 1 on-own	437.5	80% HRmax	1750
11–15	3 supervised, 1 on-own	500	80% HRmax	2000

HRmax—heart rate maximum (as determined during maximal effort exercise test).

**Table 2:** Baseline characteristics.

	AM (*n* = 18)	PM (*n* = 15)
Age (years)	40.8 ± 8.4	36.4 ± 10.8
BMI (kg/m^2^)	30.0 ± 3.9	30.6 ± 4.7
Female (*n*, %)	14, 78%	9, 60%
Race		
White (*n*, %)	16, 89%	12, 80%
Black	1, 6%	2, 13%
Other	1, 6%	1, 7%
Ethnicity		
Hispanic	3, 17%	8, 53%
Non-Hispanic	15, 83%	7, 47%

Data are reported as mean ± SD or *n*, (%).

**Table 3:** Exercise intervention adherence.

Week	Attendance (Sessions/Week)		Completed Exercise EE (kcal/Week)		Completed Intensity (% HRmax)		Exercise Prescription (min/Week)	
	AM	PM	AM	AM	AM	PM	AM	PM
1–2	3.9 ± 0.3	3.6 ± 0.6	727 ± 64	700 ± 108	72 ± 1	72 ± 1	136 ± 25	142 ± 33
3–4	3.9 ± 0.2	3.4 ± 0.9	994 ± 65	938 ± 144	72 ± 2	72 ± 2	181 ± 33	189 ± 44
5–6	3.9 ± 0.3	3.8 ± 0.5	1165 ± 111	1154 ± 157	77 ± 1	76 ± 1	170 ± 25	170 ± 38
7–8	3.8 ± 0.6	3.3 ± 0.7	1298 ± 141	1190 ± 215	77 ± 1	76 ± 3	212 ± 32	212 ± 47
9–10	3.6 ± 0.4	3.4 ± 0.7	1641 ± 225	1689 ± 252	81 ± 1	80 ± 2	218 ± 33	200 ± 45
11–15	3.2 ± 0.8	3.3 ± 0.7	1601 ± 340	1696 ± 328	80 ± 2	79 ± 5	249 ± 37	229 ± 52

**Table 4:** Change in energy expenditure and energy intake.

	Baseline	Week 15	Change
TDEE (kcal/day)			
AM	2231 ± 341	2453 ± 391	222 ± 399
PM	2548 ± 574	2638 ± 536	90 ± 150
Non-Exercise EE (kcal/day)			
AM	750 ± 316	690 ± 294	−60 ± 432
PM	948 ± 277	747 ± 228	−200 ± 152
Resting EE (kcal/day)			
AM	1457 ± 233	1477 ± 206	20 ± 117
PM	1600 ± 385	1605 ± 424	4 ± 127
Energy Intake (kcal/day)			
AM	2231 ± 341	2330 ± 367	99 ± 198
PM	2548 ± 574	2527 ± 615	−21 ± 156

## Data Availability

Data are available upon request to seth.creasy@cuanschutz.edu.
